# SMARCA4-Deficient Undifferentiated Tumor: A Rare Malignancy With Distinct Clinicopathological Characteristics

**DOI:** 10.7759/cureus.30708

**Published:** 2022-10-26

**Authors:** Ghassan Al-Shbool, Hari Krishnan Nair

**Affiliations:** 1 Hematology and Medical Oncology, Saint Louis University School of Medicine, St Louis, USA

**Keywords:** sarcomatoid carcinoma, smarca4-dut, immunotherapy, chemotherapy, swi/snf, carcinosarcoma, smarca4, thoracic, sarcoma

## Abstract

Switch/sucrose non-fermentable-related, matrix-associated, actin-dependent regulator of chromatin, subfamily A, member 4-deficient undifferentiated tumor (SMARCA4-dUT) is a rare malignancy due to inactivating mutations in the SMARCA4 gene of the switch/sucrose non-fermentable (SWI/SNF) chromatin remodeling complex. These are aggressive malignancies presenting predominantly in male smokers in their fifth and sixth decades of life with nonspecific respiratory symptoms. Most patients have metastatic disease on presentation. The pattern of metastasis is similar to carcinomas, and the most common metastatic sites are lymph nodes, bones, and adrenal glands. Histologically, these tumors can be either entirely sarcomatoid or with mixed features of sarcoma and carcinoma, with extensive necrosis and high mitotic activity. Immunohistochemistry demonstrates negative expression of keratin and claudin-4, and tumor cell nuclei characteristically lack expression of Brahma-related gene-1 (BRG1). No standard treatment guidelines have been established due to the relative rarity of these tumors, and systemic chemoimmunotherapy has demonstrated benefit in some cases. We report two cases of SMARCA4-dUT with their clinical course and management to provide a perspective on the behavior of these tumors in a Western population cohort.

## Introduction

The switch/sucrose non-fermentable (SWI/SNF) complex is an adenosine triphosphate (ATP)-dependent chromatin-remodeling complex that regulates protein transcription and cellular differentiation [1,2]. Brahma-related gene-1 (BRG1) protein, encoded by the switch/sucrose non-fermentable-related, matrix-associated, actin-dependent regulator of chromatin, subfamily A, member 4-deficient undifferentiated tumor (SMARCA4-dUT) gene located on the short arm of chromosome 19, is an essential part of this complex [3,4]. SMARCA4 inactivation and SWI/SNF mutations have been reported in 20% of several types of malignancies from various sites, such as the uterus, gastrointestinal tract, and lungs, and portends an aggressive clinical course and poor prognosis [5-7].

In 2015, a new entity called SMARCA4-deficient undifferentiated tumor (SMARCA4-dUT) was reported in the medical literature, characterized by a high-grade thoracic malignancy that demonstrated an inactivating mutation causing loss of SMARCA4 expression [8,9]. Subsequently, several case reports confirmed the histopathological and immunohistochemical features, clinical manifestations, and natural history of this aggressive malignancy that predominantly affects young men who are smokers [3,6,10]. In this report, we describe the clinical course of two patients with SMARCA4-dUT to provide further insight into the clinical, immunohistochemical, and genetic sequencing characteristics of these tumors in the Western population.

## Case presentation

Case 1

**Figure 1:**
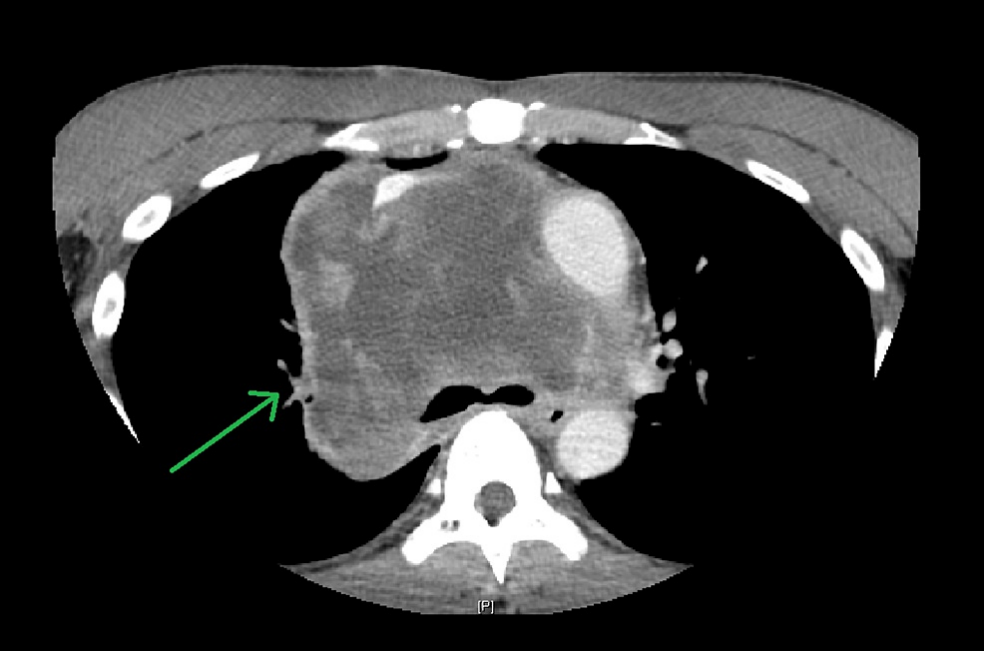
Computed tomography (CT) of the chest with intravenous contrast demonstrating the 13 cm x 10.5 cm x 11.2 cm heterogeneous mediastinal mass (green arrow) causing compression of the superior vena cava and right pulmonary artery and abutment of the adjacent vasculature

A 40-year-old male with an extensive tobacco smoking history presented with right neck swelling, unintentional weight loss, dysphagia, and dyspnea over a one-month period. Computed tomography (CT) of the neck and chest with intravenous contrast demonstrated a 13.0 cm x 10.5 cm x 11.2 cm infiltrative heterogenous mediastinal mass extending to the base of the neck on the right side, medial to the sternocleidomastoid muscle causing superior vena cava and right pulmonary artery compression as well as the abutment of the ascending aorta, aortic arch, cervical artery, brachiocephalic artery, right subclavian artery, right common carotid artery, and origin of the right vertebral artery (Figure 1). The mass caused the displacement of the local vasculature and partially encased the midthoracic trachea causing tracheal stenosis at the level of the carina, and abutment of the esophagus with radiological evidence of emphysema. CT of the abdomen with intravenous contrast demonstrated multiple peritoneal lesions concerning for metastasis.

A CT-guided core biopsy of the neck mass demonstrated a pleomorphic, high-grade, malignant neoplasm with brisk mitotic activity including frequent atypical forms. The immunohistochemistry and molecular testing results (performed by a tissue-based 648-gene next-generation deoxyribonucleic acid (DNA) sequencing panel) are shown in Table 1.

**Table 1:** Results of immunohistochemistry and molecular analysis performed on the tumor tissue

Immunohistochemistry	Molecular analysis
Vimentin: Strongly and diffusely positive	SMARCA4; p.E431* stop gain-loss of function (LOF) (63.9%)
Cell adhesion molecule (CAM) 5.2: negative	Transformation-related protein 53 (TP53); p.T125T splice region variant-LOF (59.9%)
S100: negative	Fanconi anemia complementation group A (FANCA); p.Q436* stop gain-LOF (51.7%)
Cluster of differentiation (CD) 45: negative	Retinoblastoma protein 1 (RB1); p.ME208* stop gain-LOF (41.9%)
CD30: negative	Low-density lipoprotein receptor-related protein 1B (LRP1B); p.R1652* stop gain-LOF (41.1%)
CD20: negative	Tumor mutational burden 12.1 m/MB (91st percentile)
Sex-determining region Y-box transcription factor 10 (SOX10): negative	Microsatellite instability status: stable
Calretinin: negative	Programmed death-ligand 1 (PD-L1) (28-8 and 22C3) expression: <1%
Desmin: negative	
Brahma-related gene-1 (BRG1): absent expression in tumor nuclei	

A multidisciplinary team discussion involving medical oncology, radiation oncology, otolaryngology, thoracic surgery, and pulmonology services was held, and based on consensus, the patient was initiated on palliative radiation therapy for symptomatic management (20 Gy in five fractions), followed by systemic combination chemoimmunotherapy with carboplatin, paclitaxel, and pembrolizumab. He received four cycles of systemic treatment. The further clinical course was complicated by persistent cachexia and weight loss, acute left peroneal deep venous thrombosis, and spontaneous pneumothorax. Interval CT of the chest, abdomen, and pelvis with intravenous contrast demonstrated stable disease without evidence of progression, however, the patient opted to stop further systemic chemotherapy, and hence treatment was switched to pembrolizumab maintenance alone for one further cycle. Two weeks after his first cycle of single-agent pembrolizumab, he presented to the emergency department with dyspnea and altered mental status, was noted to be hypoxic requiring supplemental oxygen, and based on discussion with family, his code status was switched to comfort measures only (CMO), and the patient expired shortly thereafter.

Case 2

**Figure 2:**
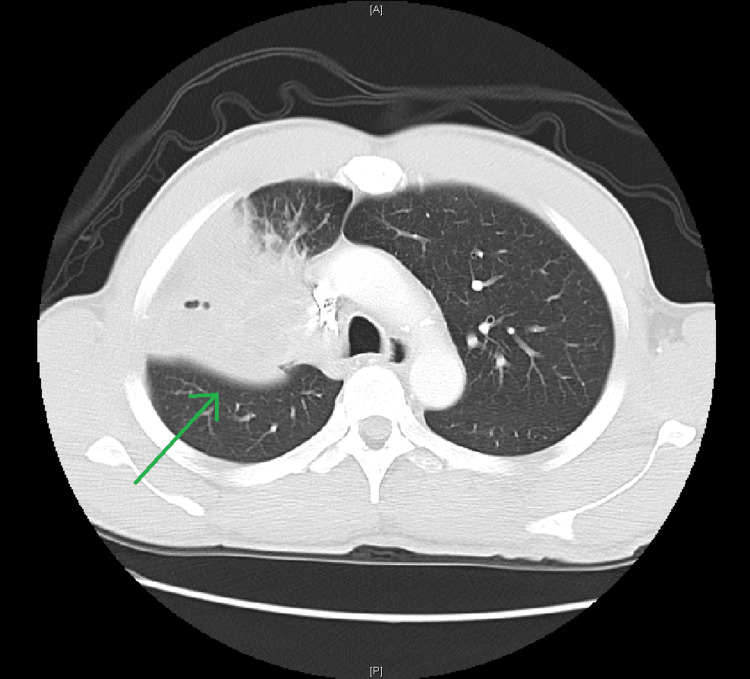
Computed tomography (CT) chest with intravenous contrast demonstrating the large suprahilar hypoattenuating lung mass (green arrow) causing right upper lobe bronchus occlusion and right upper lobe pulmonary artery narrowing

A 48-year-old male with an extensive tobacco smoking history presented to the emergency room with right-sided pleuritic chest pain. CT of the chest with intravenous contrast demonstrated a right suprahilar hypoattenuating lung mass measuring 4 cm with abrupt occlusion of the right upper lobe bronchus and narrowing of the right upper lobe pulmonary artery branches (Figure 2).

**Figure 3:**
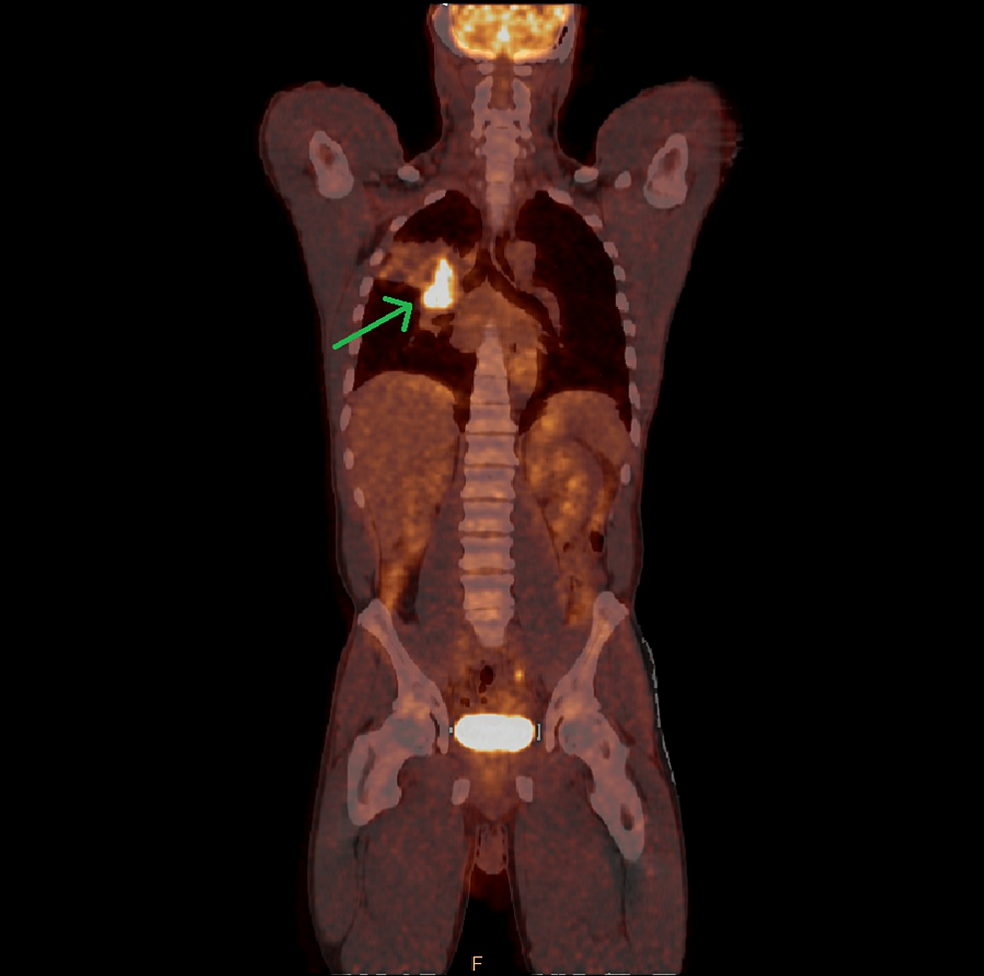
Whole-body 18F-fluorodeoxyglucose positron emission tomography (FDG-PET)/CT demonstrating the FDG-avid right suprahilar mass (green arrow) with a maximum standardized uptake value (SUVmax) of 22.1

Whole-body 18F-fluorodeoxyglucose positron emission tomography (FDG-PET)/CT was obtained, demonstrating a large, metabolically avid right hilar mass with a few enlarged subcentimeter right paratracheal and subcarinal lymph nodes (Figure 3).

Endobronchial ultrasound with final needle aspiration of lymph node station 11R was positive for malignancy, with cytology reporting non-small cell carcinoma while cytology from lymph node station 7 was negative for malignancy. A transbronchial biopsy was obtained that demonstrated bronchial mucosa infiltrated by a high-grade, non-small cell neoplasm with pleomorphic features and focal rhabdoid morphology. The immunohistochemistry results are shown in Table 2.

**Table 2:** Results of immunohistochemistry performed on the tumor tissue

Immunohistochemistry
Cytokeratin (CK) 5/6: Focally positive
Thyroid transcription factor 1 (TTF-1): Negative
Napsin A: Negative
P40: Negative
BRG1: Absent expression in tumor nuclei

A multidisciplinary team discussion involving the medical oncology, radiation oncology, thoracic surgery, and pulmonology services was held, and the patient was staged using the American Joint Committee on Cancer (AJCC) 8th edition of the Tumor Node Metastasis (TNM) classification as stage IIIA (pT4pN1M0). Treatment was initiated with concurrent chemoradiotherapy using carboplatin and paclitaxel every three weeks concurrently with 60 Gy radiation in 30 fractions for a total of five cycles. Restaging CT of the chest, abdomen, and pelvis with intravenous contrast demonstrated stable disease in the lung but with new lesions in the left adrenal gland, which was pathologically confirmed by core needle biopsy and treated with stereotactic body radiation therapy (SBRT). Treatment was then switched to systemic therapy with carboplatin, pemetrexed, and pembrolizumab every three weeks for four cycles, followed by continuous maintenance with pemetrexed and pembrolizumab. The lung lesions have remained stable over 15 months of therapy. However, he was found to have new oligometastatic lesions in the left axillary lymph node, which was treated with SBRT (30 Gy in 3 fractions), and in the right paraspinal muscle, which was again treated with SBRT (35 Gy in 5 fractions) while continuing maintenance systemic therapy. The patient is currently faring well clinically and has tolerated treatment well without significant toxicities.

## Discussion

Background

The SMARCA4 gene is a central component of the SWI/SNF chromatin remodeling complex that regulates DNA accessibility by mobilizing nucleosomes [1,2]. Inactivating mutations in different components of this complex have been reported in various malignancies over the last decade [3,5,9,11,12]. While SMARCA4 is considered a tumor suppressor gene, both the loss of protein expression as well as protein upregulation have been associated with neoplasia [13,14]. The gene was initially described in pediatric malignant rhabdoid tumors (MRT) and subsequently identified in small cell carcinomas of the ovary, hypercalcemic type (SCCOHT), and other tumor types, such as non-small cell lung carcinomas (NSCLC), mainly lung adenocarcinomas [15,16].

SMARCA4-dUT was described initially as an undifferentiated tumor of round cell or rhabdoid morphology characterized by SMARCA4 mutations with concomitant loss of expression [9]. The phenotypic expression profile was similar to MRT and SCCOHT and distinct from NSCLC, indicating a distinct type of thoracic sarcoma [3,6]. However, the tumor demonstrates certain carcinoma-like features, such as a strong association with smoking and emphysema, a high incidence of metastasis, and frequent TP53 mutations, in addition to having the smoking-related mutations seen in NSCLC, such as KRAS, KEAP1, and STK11, and the focal expression of NSCLC markers such as TTF1 and p40 [6,9].

Demographics

Based on cases reported in existing medical literature, most patients present in their fifth and sixth decades of life. The patients are predominantly male with a history of extensive smoking and with radiological evidence of emphysematous changes in the lungs [6,8,9]. The malignancy usually presents with nonspecific upper and lower respiratory symptoms from mechanical effects due to the large mediastinal mass with extensive thoracic invasion [6,8,10]. Most patients are stage IV at diagnosis with a metastatic pattern akin to carcinomas. The metastatic sites are predominantly the lymph nodes, bones, and adrenal glands. Brain metastasis, however, is of rare incidence in these tumors [3,6,8-10]. In both the cases we described, the patients were male, in their fifth decade, with a long-term history of smoking, and presented with extensive mediastinal involvement.

Pathology

Histopathologically, the tumor can appear entirely sarcomatoid or with combined features of sarcoma and carcinoma [10]. Sarcomatoid areas are characterized by monotonous round cell morphology, prominent nucleoli, and mild pleomorphism, with extensive necrosis and very high mitotic activity [6,8-10]. A mixture of rhabdoid cells with distinctive hyaline cytoplasmic inclusions has been reported in some cases [3,6,10]. Immunohistochemistry studies usually demonstrate negative expression of SMARCA2, keratin, and claudin-4 [6,9,10,15]. While there is a positive expression of vimentin and synaptophysin, TTF1 and p40 are weak and focally expressed [17]. Stem cell markers, such as Sal-like protein 4 (SALL4) and CD34, and other markers, such as CD99 and switch/sucrose non-fermentable related, matrix associated, actin dependent regulator of chromatin, subfamily B, member 1 (SMARCB1), are also expressed in some cases [6,8]. The Ki-67 proliferation rate is consistently high around 70% [6,10,18]. Features of carcinoma could be expressed focally in some cases, with either a gradual or abrupt shift to positive keratin and claudin-4 markers [10,17,19]. Molecular testing generally demonstrates a high tumor mutation burden with mutations in SMARCA4 and TP53 in addition to mutations commonly seen in smoking-associated NSCLC such as serine/threonine kinase 11 (STK11), Kelch-like ECH-associated protein 1 (KEAP1), and Kirsten rat sarcoma virus (KRAS), including KRAS G12C. These mutations are usually absent in sarcomas [3,6,9].

The diagnosis is complex and requires an adequate tissue sample, with a review by an expert pathologist [20]. Sometimes, the malignancy may be labeled as a tumor of unknown primary or undifferentiated carcinoma [10], and the presence of neuroendocrine markers and high Ki-67 may lead to misdiagnosis [6,9,20]. Both cases reported by us required referral to an expert pathologist at a referral institution for confirmatory diagnosis.

Treatment

Due to the rarity of SMARCA4-dUT, there are no standard guidelines for the treatment of these tumors, which reiterates the necessity of clinical trials to establish a first-line treatment regimen [6,10]. The tumors are considered chemotherapy-resistant, with prior studies demonstrating the limited benefit of short duration with adriamycin and ifosfamide [6], and more recent studies demonstrating some benefit with pembrolizumab and nivolumab [8,20]. As most patients are stage IV at the time of presentation, systemic chemoimmunotherapy followed by maintenance therapy has been used in most cases reported [8,20]. Novel agents and new therapeutic approaches, such as enhancer of zeste homolog 2 (EZH2) inhibitors and cyclin-dependent kinase (CDK)4/6 inhibitors, are under investigation, with a developing interest in the usage of immune checkpoint inhibitors given the high tumor mutational burden exhibited by these tumors [8,10,20]. The prognosis is usually dismal, with overall survival ranging from weeks to months, but recent cases reported a prolonged response with immunotherapy [20].

## Conclusions

SMARCA4-dUT is a rare malignancy seen predominantly in male smokers caused by inactivating mutations in the SMARCA4 gene of the SWI/SNF chromatin remodeling complex. These tumors present typically as a large mediastinal mass and are invariably metastatic on presentation. Histopathologically, they exhibit features of both sarcomas and carcinomas to varying degrees, and they characteristically lack expression for BRG1 on immunohistochemistry, which establishes the diagnosis. Standard guidelines for treatment are limited due to the rarity of these tumors, however, there are reports of good responses to chemoimmunotherapy. In our case series reported to provide a perspective on the management of these patients in a Western population cohort, both patients received combination systemic treatment with chemoimmunotherapy and showed good disease control and duration of benefit.
